# Immunocytes do not mediate food intake and the causal relationship with allergic rhinitis: a comprehensive Mendelian randomization

**DOI:** 10.3389/fnut.2024.1432283

**Published:** 2024-09-27

**Authors:** Zhi-qiang Zhang, Jing-yang Li, You-wei Bao, Yu-Qi Song, Dong-xu Song, Cheng Wang, Xin-hua Zhu

**Affiliations:** ^1^Department of Otorhinolaryngology, Head and Neck Surgery, The Second Affiliated Hospital, Jiangxi Medical College, Nanchang University, Nanchang, China; ^2^Department of Clinical Medicine, The First School of Clinical Medicine, Nanchang University, Nanchang, China; ^3^Department of Gastrointestinal Surgery, Union Hospital, Tongji Medical College, Huazhong University of Science and Technology, Wuhan, China; ^4^Department of Orthopaedics, Union Hospital, Tongji Medical College, Huazhong University of Science and Technology, Wuhan, China; ^5^Department of Critical Care Medicine, Medical Center of Anesthesiology and Pain, The First Affiliated Hospital, Jiangxi Medical College, Nanchang University, Nanchang, China

**Keywords:** Mendelian randomization, allergic rhinitis, B cells, granulocytes, food intake

## Abstract

**Background:**

Observational studies indicate a correlation between food intake and allergic rhinitis. The potential interplay between the immune system and allergic rhinitis might contribute causally to both food intake and allergic rhinitis, providing promising therapeutic avenues. However, elucidating the causal relationship and immune-mediated mechanisms between food intake and allergic rhinitis remains a pending task.

**Methods:**

We utilized a two-sample Mendelian randomization (MR) methodology to explore the causal relationship between food intake and allergic rhinitis. Furthermore, we investigated the potential causal relationship of immune cell signals with allergic rhinitis, as well as the potential causal relationship between food intake and immune cell signals. Moreover, employing both two-step Mendelian randomization and multivariable Mendelian randomization, we delved into the mediating role of immune cell signals in the causal relationship between food intake and allergic rhinitis. Leveraging publicly accessible genetic datasets, our analysis encompassed 903 traits, comprising 171 food intake features, 731 immune cell features, and one trait related to allergic rhinitis.

**Result:**

We found causal relationships between seven types of food intake and allergic rhinitis, as well as between 30 immune cell phenotypes and allergic rhinitis. Furthermore, our two-step Mendelian randomization analysis and multivariable Mendelian randomization analysis indicate that immune cells do not mediate the causal relationship between food intake and allergic rhinitis.

**Conclusion:**

To the best of our knowledge, we are the first to incorporate a large-scale dataset integrating immune cell features, food intake features, and allergic rhinitis into Mendelian randomization analysis. Our research findings indicate that there are causal relationships between six types of food intake and allergic rhinitis, as well as between 30 immune cell phenotypes and allergic rhinitis. Additionally, immune cells do not mediate these relationships.

## 1 Introduction

Previous studies have established a causal link between food intake and allergic rhinitis, as evidenced by the findings reported in these studies (1–6). Pathophysiologic changes occurring in the organism as a result of food intake involve epithelial barrier dysfunction and dysregulation of the immune response (7), it ultimately leads to Th1/Th2 dysregulation (8), which affects immune cell activity (9) and immune regulation (10) as well as leading to changes in interleukins in the body (11), which leads to the development of allergic rhinitis. In addition, numerous studies have shown that food intake leads to nasal pathophysiological changes in patients/mice, such as allergic symptoms, itchy eyes, sneezing, runny nose, and sleep disturbances (3, 12–14), which May be caused by immunoglobulin E (IgE)-mediated hypersensitivity to incoming allergens (15, 16). The relationship between food intake and allergic rhinitis has been reported in several clinical studies. Pang et al. (17) showed a possible association between seafood intake and allergic rhinitis through a retrospective study including (18, 19), in addition to Yoshihiro Miyake et al. (20) demonstrated an association between *β*-carotene-containing food intake and allergic rhinitis, and Farchi et al. (21) analyzed a study and came to a similar conclusion that nut and butter intake was associated with the development of allergic rhinitis. More interestingly, previous studies have shown that maternal intake of meat or n-6 polyunsaturated fatty acid-rich foods during pregnancy May increase the risk of allergic rhinitis in the fetus at birth (5, 22). Despite this, the precise mechanism underlying this causal relationship, particularly whether immune cells play a mediating role, remains enigmatic. Consequently, the primary objective of our study was to investigate whether immune cells function as mediators in the association between food intake and allergic rhinitis.

Food intake occurs in the context of environmental stimuli known as ambience (23), a complex physiological process necessary for survival. This process is influenced by assimilation mechanisms and the compatibility of food with a person’s appetite (24). When individuals engage in eating, they consider various factors, including the timing, type, and quantity of food. Habit, convenience, or opportunity often play a role in determining when to eat, rather than solely physiological need (25). Additionally, a myriad of external factors impact food intake and choices. These factors encompass the social and physical environment, including the presence of others, sounds, temperatures, odors, colors, time of day, and distractions. Notably, the temperature, odor, and color of food itself can have distinct effects on food intake and preferences (23). When given a choice of choosing foods, individuals typically base their decisions on pleasure and past experiences (25). However, contrary to the long-held belief that eating is a homeostatic behavior in the body, recent evidence suggests that it is not solely an automatic response to energy deficiency. Instead, food intake can be viewed as a long-term, integrative response aimed at maintaining stored energy levels in adipocytes (25). This integrated response involves complex interactions between various physiological systems and environmental cues. To summarize, food intake is a multifaceted process influenced by both internal physiological mechanisms and external environmental factors. Understanding these interactions is crucial for comprehending eating behaviors and their implications for health and well-being.

Allergic rhinitis, a common chronic allergic inflammatory disease, typically persists throughout a person’s lifetime (26, 27). It results from a type I hypersensitivity reaction in the nasal mucosa upon exposure to airborne allergens such as grass pollen, house dust mites, and animal dander. The hallmark symptoms of this condition include nasal congestion, watery rhinorrhea, pruritus, and paroxysmal sneezing (28). Notably, allergic rhinitis is associated with elevated immune cell recruitment (29). Previous studies have demonstrated a reduced quality of life among patients with allergic rhinitis, particularly among adults compared to adolescents (30). Epidemiological studies reveal that approximately 20 to 30% of adults and up to 40% of children are affected by this condition (31). The symptoms of allergic rhinitis can significantly impact a patient’s quality of life, causing frequent sleep disturbances and leading to impaired performance at work and school (32). Furthermore, this condition places a significant health burden on individuals, affecting their quality of life and associating with severe comorbidities such as asthma (33). Additionally, allergic rhinitis has a considerable impact on the healthcare economy, affecting education, productivity, and the utilization of healthcare resources (33).

The immune system, a dynamic and integrated network, primarily comprises a diverse array of immune cells distributed throughout the body. These cells work in concert to maintain tissue homeostasis and elicit protective immunity against external threats (34). Originating from stem cells in the bone marrow, these immune cells differentiate into multiple lineages, including granulocytes, macrophages, dendritic cells (DCs), T cells, B cells, and natural killer cells (NK cells) (35). They can respond to alterations in the internal and external environment, regulating immunity and safeguarding the host from pathogens, foreign substances, and malignant tumors (36). However, dysregulation of the immune system can result in suppressed or hyperactive immune cells, thereby influencing the initiation and progression of various diseases (37–39).

Dietary habits have long been identified as a potential environmental risk factor for the increasing incidence of autoimmune diseases (40, 41). Notably, high salt intake has been demonstrated to promote the differentiation of proinflammatory T helper cell 17 (Th17) (42, 43). Similarly, high glucose intake also drives Th17 cell differentiation via the activation of transforming growth factor-*β* (TGF-β) (44). Consequently, the alterations in immune cells induced by dietary intake cannot be overlooked.

Recent studies have shown a high correlation between immune cells and allergic rhinitis (15, 45). For instance, Fokkens et al. (46) demonstrated that Langerhans cells play a crucial role in the nasal mucosa of allergic patients during allergic episodes, while Salib et al. (47) established a link between allergic rhinitis and mast cells. Furthermore, other researchers have shown that eosinophil elevations can also trigger allergic rhinitis attacks (48). Notably, allergic rhinitis is accompanied by a localized accumulation of activated T-helper cells, eosinophils, and neutrophils in the affected organ upon allergen exposure (49).

The aim of our study was to investigate the association between food intake-induced allergic rhinitis and immune cell-mediated allergic rhinitis, in order to gain a better understanding of the role of immune cells in the pathogenesis of food-induced allergic rhinitis. However, contrary to our expectations, the results indicated that immune cells do not serve as mediators in food intake-induced allergic rhinitis.

## 2 Methods

### 2.1 Immunity-wide GWAS data

Summary statistics for each immunophenotype are publicly available in the GWAS Catalog, ranging from accession numbers GCST90001391 to GCST90002121. These statistics cover a total of 731 immunophenotypes, including absolute cell counts (*n* = 118), median fluorescence intensity (MFI) reflecting surface antigen levels (*n* = 389), morphological parameters (MP) (*n* = 32), and relative cell counts (*n* = 192). These features span various developmental stages and cell types of immune cells. The original GWAS for immunophenotypes utilized data from 3,757 European individuals across non-overlapping cohorts. The instrumental variable (IV) significance level for each immunophenotype was set at 1 × 10^(−5). We pruned these SNPs using a linkage disequilibrium (LD) r2 threshold of <0.1 within a 500 kb distance.

### 2.2 Food intake GWAS data

Summary statistics for each food intake phenotype can be accessed publicly in the GWAS Catalog. The significance level for each instrumental variable (IV) was set at 1 × 10^(−5). We pruned these SNPs using a linkage disequilibrium (LD) r2 threshold of <0.001 within a 10,000 kb distance. The GWAS ID corresponding to each of the 171 food intake phenotypes can be found in Supplementary Table S1.

### 2.3 Allergic Rhinitis GWAS data

The Allergic Rhinitis GWAS data were sourced from the public GWAS Catalog, with the GWAS ID being ebi-a-GCST90018792. We pruned these SNPs using a linkage disequilibrium (LD) r2 threshold of <0.001 within a 10,000 kb distance.

### 2.4 Statistical analysis

MR analyzes the causal relationship between food intake and AR and explores whether immune cells act as mediators to mediate this process. In the absence of horizontal pleiotropy, the inverse variance weighting (IVW) method can be the main method for analyzing causality in TSMR analyses (50). Prior to this, we implemented Cochrane’s Q test to assess heterogeneity between IVs. If heterogeneity was detected (*p* < 0.05), a random-effects IVW model could provide more conservative estimates; otherwise, a fixed-effects IVW model would be used (51). Other MR analysis methods, including weighted median estimator (WM) and MR-Egger regression (52), can complement the IVW approach and provide wider confidence intervals (53). These three MR methods for causal inference have their own modeling assumptions. The IVW method is applicable in the absence of horizontal multinomiality (50); the WM method assumes that less than 50% of IVs are horizontally multinomial (54); and MR-Egger regression assumes that more than 50% of IVs are affected by horizontal multinomiality (52).

We considered a possible causal relationship between food intake and allergic rhinitis if the results of the MR analysis were nominally significant (*p* < 0.05) (55). Results were considered reliable if a significant causal relationship between food intake and outcome was determined by two or more MR methods (56).

The existence of horizontal pleiotropy May challenge the second MR hypothesis; therefore, we adopted various methods to monitor possible horizontal pleiotropy. Specifically, the *p*-value of the MR-Egger intercept test and MR pleiotropy residual sum and outlier (MR-PRESSO) global test can be used to assess the existence of horizontal pleiotropy, and *p* < 0.05 was considered statistically significant (56, 57). The MR-PRESSO outlier test can adjust horizontal pleiotropy by detecting and removing outliers (58), and the number of distributions in the MR-PRESSO analysis was set to 1,000 (59).

Additionally, we conducted a leave-one-out sensitivity analysis of the identified significant results to determine whether the causal relationship of the MR analysis was caused by a single SNP (60). Finally, a reverse MR analysis was performed between allergic rhinitis and the identified significant food intake using positive MR analysis to examine whether a reverse causal association existed. The reverse MR procedure was the same as that for the above MR analysis. TSMR analyses were performed using the ‘MR’ (version 0.5.6) in R software (version 4.2.1).

## 3 Results

### 3.1 Causal effect between food intake and allergic rhinitis

**Figure 1:**
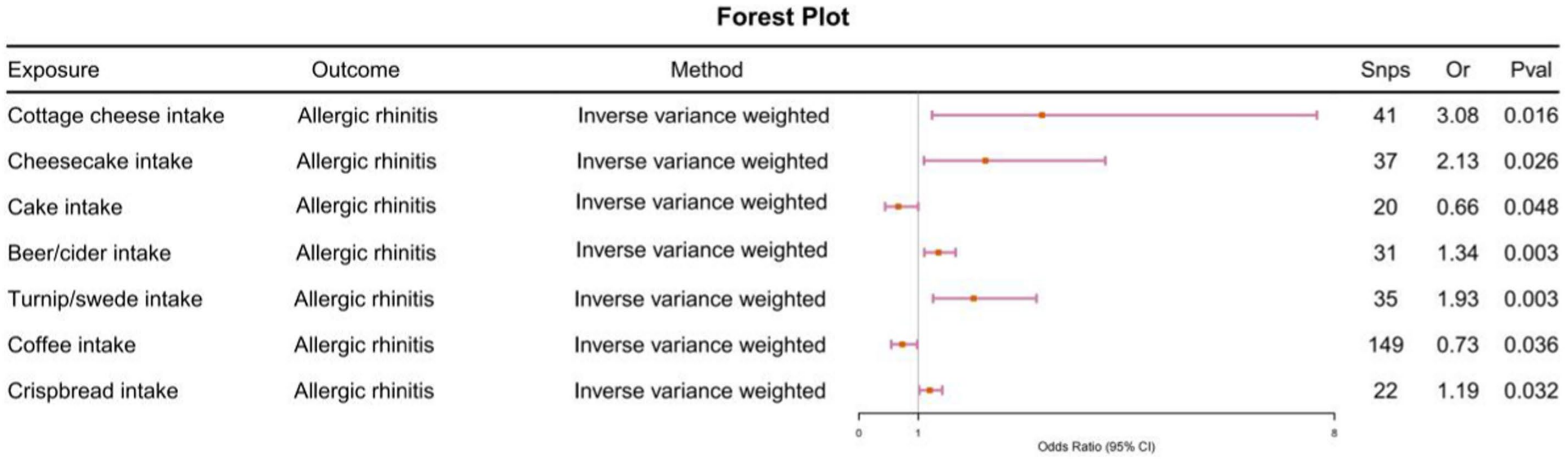
Using Inverse variance weighted methods to analyze the causal association between food intake and allergic rhinitis.

We evaluated whether 171 types of food intake (Supplementary Table S1) are causally related to allergic rhinitis. We primarily utilized the IVW method, and the results indicated causal associations between allergic rhinitis and 7 types of food intake. Cottage cheese intake was found to be causally associated with allergic rhinitis (odds ratio [OR] = 3.08, 95% confidence interval [CI] = 1.23–7.71, *p*-value <0.05). Cake intake showed a causal association with allergic rhinitis (odds ratio [OR] = 0.66, 95% confidence interval [CI] = 0.44–0.99, *p*-value <0.05). Cheesecake intake demonstrated a causal association with allergic rhinitis (odds ratio [OR] = 2.12, 95% confidence interval [CI] = 1.09–4.14, *p*-value <0.05). Beer/cider intake was causally associated with allergic rhinitis (odds ratio [OR] = 1.34, 95% confidence interval [CI] = 1.10–1.62, *p*-value <0.05). Turnip/swede intake showed a causal association with allergic rhinitis (odds ratio [OR] = 1.93, 95% confidence interval [CI] = 1.24–2.98, *p*-value <0.05). Coffee intake was causally associated with allergic rhinitis (odds ratio [OR] = 0.73, 95% confidence interval [CI] = 0.54–0.98, *p*-value <0.05). Crispbread intake demonstrated a causal association with allergic rhinitis (odds ratio [OR] = 1.19, 95% confidence interval [CI] = 1.01–1.40, *p*-value <0.05) (Figure 1). Horizontal pleiotropy was assessed using the MR Egger method. The *p*-values for the MR-Egger regression intercepts were all greater than 0.05, indicating no evidence of horizontal pleiotropy (Supplementary Table S2). Heterogeneity tests were conducted using both the Inverse Variance Weighted and MR Egger methods, with p-values exceeding 0.05 for the other 6 types of food intake, besides coffee intake, suggesting no heterogeneity present (Supplementary Table S3). The LOO analysis revealed a consistent trend for all SNPs included in our analysis, and scatter plots further demonstrated the robustness of our study results (Supplementary Figure S1). Subsequently, we performed reverse Mendelian randomization analysis with allergic rhinitis as the exposure and food intake as the outcome (Supplementary Table S4). The results indicated no causal relationships (*p*-values >0.05) between them. Therefore, our analysis concludes that the causal relationships between the 6 types of food intake and allergic rhinitis remain reliable.

### 3.2 Causal effect between immunophenotypes and allergic rhinitis

To investigate whether immune cell phenotypes have a causal relationship with allergic rhinitis, we conducted a two-sample Mendelian randomization analysis with 731 immune cell phenotypes as exposures and allergic rhinitis as the outcome. The results revealed that 30 immune cell phenotypes were causally associated with allergic rhinitis (*p*-value <0.05) (Supplementary Table S5). Subsequently, we performed reverse Mendelian randomization analysis to further validate our findings, which showed non-significant results (*p*-value >0.05) (Supplementary Table S6).

### 3.3 Causal relationship between food intake and allergic rhinitis mediated by immune phenotypes

**Figure 2:**
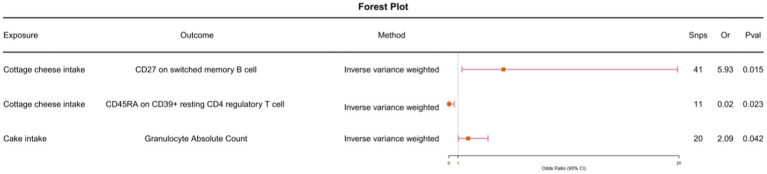
Using Inverse variance weighted methods to analyze the causal association between food intake and immunophenotype.

To further investigate whether immune cells mediate the causal relationship between food intake and allergic rhinitis, we conducted Mendelian randomization analysis with 30 immune cell features that were causally associated with allergic rhinitis as outcomes and food intake as exposures. Cottage cheese intake and memory B cells (odds ratio [OR] = 5.93, 95% confidence interval [CI] = 1.41–24.83, *p*-value <0.001). Cottage cheese intake and regulatory T cells (odds ratio [OR] = 0.02, 95% confidence interval [CI] = 0.0006–0.59, p-value <0.001). Cake intake and granulocytes (odds ratio [OR] = 2.09, 95% confidence interval [CI] = 1.03–4.25, *p*-value <0.001) (Figure 2). We conducted heterogeneity tests and horizontal pleiotropy tests, with all resulting *p*-values being greater than 0.05. This indicates the absence of heterogeneity and horizontal pleiotropy (Supplementary Tables S7, S8).

**Figure 3:**
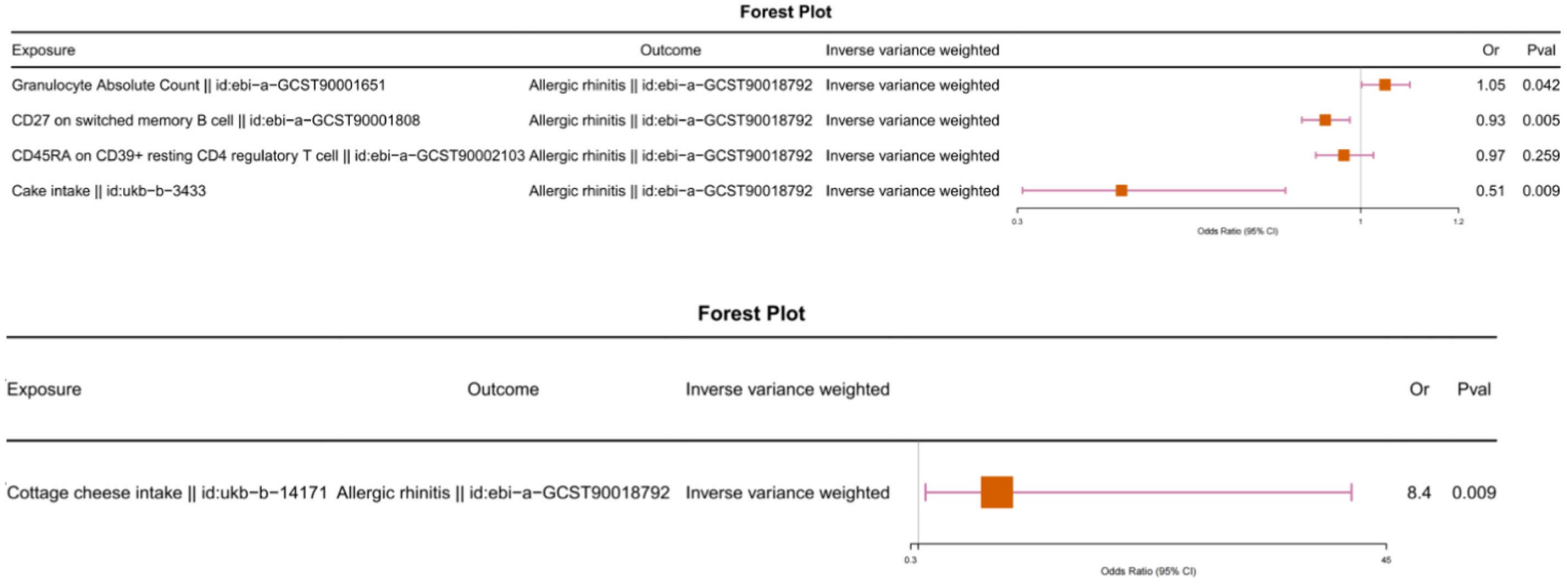
Multivariable Mendelian randomization analysis with food intake and immunophenotype as exposures and allergic rhinitis as the outcome.

We observed that the causal relationship between cottage cheese intake and memory B cells, as well as between memory B cells and allergic rhinitis, had different directions (Figure 2; Supplementary Table S5). Similarly, the causal relationship between cake intake and granulocytes, and between granulocytes and allergic rhinitis, exhibited different directions. Furthermore, we conducted multivariable Mendelian randomization analysis, which further corroborated that immune cells do not serve as intermediaries in mediating the causal relationship between food intake and allergic rhinitis (Figure 3).

## 4 Discussion

To our knowledge, we are the first to explore the causal relationships between immune phenotypes and allergic rhinitis, as well as between food intake and allergic rhinitis. We also investigated the potential mediating effects. For the first time, we incorporated over 900 traits into Mendelian randomization analyses. Furthermore, we identified seven types of food intake and 30 immune cell phenotypes that have causal relationships with allergic rhinitis. However, immune cells do not act as mediators in the causal relationship between food intake and allergic rhinitis.

### 4.1 Cottage cheese intake and allergic rhinitis

In our study, we found an association between the intake of cottage cheese and the development of allergic rhinitis. This finding aligns with T Mulch’s study, which demonstrated that many food allergies manifest as allergic rhinitis, with cheese being one of the most common allergens (61). Furthermore, Cevızcı’s et al. (62) research indicated the presence of mites and cheese mites in cheese, which can trigger allergic rhinitis upon consumption. Cheese is an IgE-like antibody (63) that binds to high-affinity IgE receptors on mast cells and basophils, thereby mediating the development of allergic rhinitis (64).

### 4.2 Cake intake and allergic rhinitis

Interestingly, while cake intake has been shown to have a protective effect against the development of allergic rhinitis, herbal cake specifically has been found to improve symptoms in patients with moderate to severe persistent allergic rhinitis (65).

### 4.3 Cheesecake intake and allergic rhinitis

Our results indicated that cheesecake intake also led to the development of allergic rhinitis. This May be attributed to the lysozyme contained in cheese, which is a potential food allergen that can sensitize individuals to develop allergic rhinitis upon consumption (66). Moreover, mites present in cheesecake (62) are a significant allergen for allergic rhinitis (67, 68).

### 4.4 Beer/cider intake and allergic rhinitis

Our analysis of five alcoholic drinks (Supplementary Table S1) showed that Beer / cider intake May play an important role in the development of allergic rhinitis (Figure 1). The existing evidence suggests that beer can cause allergic rhinitis in many people (69) and Alvarez-Cuesta et al. (70) noted that ingredients that can cause allergic diseases were detected in cider-bars, which are consistent with our findings. Notably, the occurrence of allergic rhinitis due to beer/cider ingestion May not be caused by the alcohol component but mediated by additional chemicals (polyphenols) in alcoholic beverages, which May be supported by the fact that our analysis of other similar alcohol products did not show positive results similar to those of beer/cider. Studies have shown that red wine, barley and hops contain polyphenols that inhibit COX-1 enzyme (71), Westergren et al. (72) showed that COX-1 is expressed in nasal mucosal epithelial cells in patients with allergic rhinitis (seasonal and perennial), suggesting that beer May be involved in the pathogenesis of allergic rhinitis by influencing COX-1 levels through polyphenols. However, this conclusion May be preliminary, as studies have shown that changes in COX-1 levels resulting from the ingestion of alcoholic beverages are predominantly found in red wine, beer and white wine (71). This May be partly due to the fact that different alcoholic drinks possess different fermentation methods, combined with factors such as raw materials, barrels and environment, which influence the nascent microflora and associated phenolics (73). On the other hand, from a statistical perspective, the sample size of our included data May not be enough to show the statistical efficacy of other alcoholic drinks. Therefore, more clinical studies with large center samples and high-throughput sequencing results May be needed to more carefully discuss the association of alcoholic drinks with allergic rhinitis, especially the contribution of red wine and white wine.

### 4.5 Turnip/swede intake and allergic rhinitis

Intake of turnip/swede has also been implicated in increasing the risk of allergic rhinitis. A 2008 study identified turnip as one of the most common allergens causing allergic rhinitis (74). Similarly, findings by Rhee et al. (75) showed that higher daily intake of carrots was associated with an increased risk of allergic rhinitis. Furthermore, a previous pathology report described a case where a patient with a history of persistent allergic rhinitis experienced anaphylaxis after consuming carrots (76). The 2S albumin allergen in turnip is highly cross-reactive, and it mediates allergic disease through IgE antibodies (77).

### 4.6 Coffee intake and allergic rhinitis

Coffee intake was also shown to be a protective factor against allergic rhinitis in our findings. Previous studies have shown that polyphenols present in coffee have anti-inflammatory and anti-allergic effects (78). In addition, recent studies have reported that coffee attenuates food allergen-induced airway hyperresponsiveness and systemic allergic responses in mice, with potent immunomodulatory and anti-inflammatory effects (79).

### 4.7 Crispbread intake and allergic rhinitis

Our findings also identified crispbread intake as a risk factor for allergic rhinitis. This is due to the presence of aqueous extracts of flour in crispbread, which have been shown to cause the development of allergic rhinitis (80). Both baking powder and flour extracts consistently induce neutrophilic inflammation in a non-Toll-like receptor 4-dependent manner, resulting in allergic sensitization (81). Similarly, Moscato’s et al. (82) study demonstrated that flour allergens frequently contribute to the development of rhinitis and May exacerbate allergic airway inflammation. Similarly, Mbatchou Ngahane’s et al. (83) study concluded that allergy to flour is independently associated with allergic rhinitis symptoms.

### 4.8 Immune cells do not play a mediating role in the food intake-AR association

We also examined the causal relationship between immune cells and allergic rhinitis, revealing that a lower Plasma Blast-Plasma Cell % lymphocyte ratio is associated with a reduced risk of developing allergic rhinitis. Similarly, a lower Absolute Count of CD25+ CD45RA+ CD4 non-regulatory T cells is also associated with a reduced risk. Furthermore, cxcr3 chemokines secreted by T cells play a crucial role in allergic rhinitis (84), particularly by disrupting the Th1/Th2 balance (85). Notably, CXCR3 expression in T cells is reduced in allergic rhinitis patients at the onset of the disease (85). Additionally, Naive CD8+ T cell Absolute Count and Naive CD8+ T cell %CD8+ T cell are protective factors against allergic rhinitis. Previous research has demonstrated that CD8 Tregs can mitigate or suppress the inflammatory response in allergic rhinitis (86, 87). Furthermore, several T cell subsets and their Absolute Counts, such as Terminally Differentiated CD4-CD8- T cell %T cell, CD28+ CD45RA+ CD8dim T cell %CD8dim T cell, Terminally Differentiated CD4-CD8 - T cell Absolute Count, and CD28+ CD45RA+ CD8+ T cell Absolute Count, are also protective against allergic rhinitis. Our findings also indicate that certain CD molecules expressed on cells, including CD20 on CD20- CD38- B cells, CD27 on CD24+ CD27+ B cells, CD27 on T cells, CD27 on IgD+ CD38- unswitched memory B cells, CD27 on unswitched memory B cells, CD27 on switched memory B cells, CD3 on HLA DR+ CD4+ T cells, CD25 on CD39+ resting CD4 regulatory T cells, CD33 on CD33+ HLA DR+ CD14dim, CD33 on CD33dim HLA DR+ CD11b-, CD33 on Granulocytic Myeloid-Derived Suppressor Cells, CD39 on CD39+ activated CD4 regulatory T cells, CD4 on secreting CD4 regulatory T cells, CD4 on activated & secreting CD4 regulatory T cells, CD45RA on resting CD4 regulatory T cells, and CD45RA on CD39+ resting CD4 regulatory T cells, among others, can delay the onset of allergic rhinitis. For instance, a study conducted by Shiteng Duan in 2019 demonstrated that CD33 recruitment can attenuate IgE-mediated allergic reactions and desensitize mast cells to allergens (88), thereby slowing down the progression of allergic rhinitis. Another study revealed that adhesion facilitates the differentiation of allergic rhinitis CD4IL4 T cells through ICAM1 and E-Selectin (89), leading to the production of the anti-inflammatory factor IL4. Additionally, cell surface protein molecules like HVEM expressed on naive CD8+ T cells also serve as protective factors against allergic rhinitis. A previous investigation showed that the HVEM-NFκB pathway can effectively suppress airway smooth muscle (ASM) proliferation and inflammatory responses by modulating LIGHT (also known as TNFSF14, which mediates signaling that can lead to various inflammatory diseases and airway remodeling) (90).

However, on the contrary, we have also identified some risk cell factors for allergic rhinitis. For example, Transitional B cell lymphocyte has been associated with an increased risk of allergic rhinitis. The primary role of B cells in allergy is the production of IgE, an antibody isoform that triggers an immediate hypersensitivity reaction via a mediator released by mast cells and basophils (91). In other words, B lymphocytes can produce allergen-specific IgE antibodies that mediate allergic rhinitis (92). The results also showed that Granulocyte Absolute Count is one of the risk factors for allergic rhinitis. It has been shown that granulocyte-macrophage colony-stimulating factor (GM-CSF) is a potent pro-inflammatory cytokine, which acts as an eosinophil colony-stimulating factor involved in the onset of allergic rhinitis (93). In addition, some surface molecules have been validated to promote allergic rhinitis flare-ups, such as CD19 on IgD- CD27- B cell, CD25 on IgD+ CD38- B cell. CD19 is a B-cell specific cell surface molecule belonging to the immunoglobulin superfamily, which is expressed exclusively on B cells. It plays a key role in both B cell activation and autoimmunity (94). Furthermore, B cells expressing CD19 and CD25 can spontaneously secrete IgA, IgG, and IgM subclasses and exhibit enhanced migratory capabilities (95). Additionally, these cells secrete elevated levels of pro-inflammatory cytokines, including IL-6 and INF-*γ*, and are more effective at presenting alloantigens to CD4 T cells (96). Additionally, CD14 on CD14+ CD16+ monocytes further contributes to the development of allergic rhinitis. Previous research has demonstrated that CD14+ monocytes directly participate in attracting other immune cells to produce pro-inflammatory chemokines and are rapidly recruited to the site of attack (97).

Our study found that food intake contributes to the development of allergic rhinitis, especially ultra-processed/dairy foods (Figure 1), and this association does not appear to be directly mediated by immune cells as we routinely understand. Excessive intake of ultra-processed/dairy foods increases intestinal permeability, leading to intrinsic mucosal damage and impairment of the epithelial barrier (98, 99). Studies have shown that the food emulsifier glyceryl monolaurate has been shown to impair intestinal barrier function, leading to dysbiosis of the intestinal flora. Similar findings have been found with emulsifiers such as carboxymethyl cellulose (CMC), which perhaps increases the chance of bacterial invasion and colonization by damaging the epithelial barrier of the gut (99, 100). In addition, high levels of ultra-processed food consumption have also been shown to negatively affect the composition and specific functions of the gut microbiome through changes in gut microbial taxa (101, 102). Dysbiosis of the intestinal flora, on the one hand, will cause the bacteria colonizing the intestinal tract to stimulate the human immune system with ligands such as lipopolysaccharides, flagellin and fatty acids, which will activate the immune system and lead to the activation of naïve T-cells and the production of Th1, Th2, and Th17 (103), which will in turn affect the mast cells and other cells, which will produce degranulation changes that will lead to an increase in the production of IgE, thus leading to the onset and progression of allergic rhinitis (104, 105). On the other hand Watts (106) and Zhu et al. (107) by comparing the composition of the gut flora of AR patients with that of the normal population in an analytical study confirmed that the diversity of the gut microbiota was significantly reduced in patients with AR, with an increase in the abundance of pathogenic bacteria such as Anaplasma phylum, and a decrease in the levels of Clostridium and Aspergillus species, and a similar finding was found in the study by liu et al. (108). More importantly, intestinal epithelial cells play a crucial role in intestinal immunity as mediators linking the human immune system and colonizing bacteria (109). Immune cells such as dendritic cells and Tregs in the lamina propria of intestinal epithelial cells react with bacteria colonizing the gut (109, 110). When dysbiosis occurs in the gut causing disruption of the epithelial barrier, bacteria promote the secretion of anti-inflammatory IL-10 by macrophages, decrease mTOR kinase activity and increase the production of antimicrobial peptides (111, 112). Dendritic cells can ingest invading bacteria and undergo further antigen presentation, recruiting cytokines to maintain the function of Tregs further affecting IL-4 and IFN-*γ* levels (113). All of these contribute to the onset and development of upper airway inflammation by regulating the Th1/Th2 balance (114), and we therefore speculate that perhaps food intake-induced allergic rhinitis May be attributable to dysbiosis of the intestinal flora. We expect that future studies will give more consideration to the mediating role of gut flora in food intake-related allergic rhinitis.

Although our findings May be preliminary, the association between food intake and allergic rhinitis May be clear, attributable to the fact that we rigorously screened DNA fragments to represent traits that May not have changed during DNA replication since we were born (115). I recommend that allergic rhinitis patients and clinicians make the necessary assessments and refer to our suggestions in their daily dietary management and clinical practice. Firstly, clinicians managing patients with allergic rhinitis should try to minimize controlling their intake of cottage cheese, cheesecake, beer/cider, turnip/swede, and crispbread, and May allow them to consume cake (no cheese) or coffee to as surrogate for palatability. Furthermore, the diet of patients with allergic rhinitis should follow certain guidelines to ensure adequate nutrition and diversify the dietary structure. For dairy products such as cheese, hydrolyzed formula products can be used to slow down the allergic reaction (116). For foods containing dietary polyphenols (e.g., cocoa, coffee, tea, etc.) can be combined when in the daily diet to prevent allergies (117). In addition, patients can consume fish at least twice a week (118), and previous studies have shown that regular use of fish reduces the risk of allergic rhinitis (1, 4). What’s more, patients can increase their intake of green leafy vegetables. Previous studies have shown that regular and regular intake of consumption of green leafy vegetables significantly reduces the risk of developing allergic airway diseases such as asthma (119–121). As for alcoholic beverages, I would suggest that they should be avoided as much as possible, even though our study only showed a correlation between beer/cider and allergic rhinitis, yet it is indeed an indisputable fact that alcoholic beverages can cause nasal symptoms and airway symptoms in most of the population (122–124). Finally, in order to accurately assess the patient’s immune function to improve the diagnostic accuracy of food intake-induced AR. For AR induced by food intake, a thorough dietary investigation in the history May be essential for the diagnosis, which not only greatly improves our diagnostic accuracy, but also provides an effective aid in the treatment of the patient by cutting off the intake of suspected allergenic foods. Additionally, the 2023 International Consensus Statement on Allergy and Rhinology (125) suggests that serum allergen-specific immunoglobulin E and skin prick testing May be considered as a first-line diagnostic option and is recommended as a grade B (the highest grade). However, careful history taking combined with serum allergen-specific immunoglobulin E May be a better choice if finances permit, because serum testing is more accurate, results are more interpretable, and can strongly assist the clinician in blocking suspected allergens and selecting medications (125).

Nowadays, it is difficult to change the environment or other lifestyles, but diets remain modifiable and dietary modifications can be used to prevent or control allergic diseases. Dietary diversity is a useful indicator to describe total dietary intake. Dietary diversity (focusing on healthy foods) during pregnancy and infancy has been reported to reduce allergic outcomes in offspring (5, 126, 127). Allergen diversity and food group diversity, such as fruits and vegetables in infancy, May also reduce food allergies in children (6). Therefore, especially at present, a large number of randomized controlled studies and evidence-based medical studies are needed to provide more evidence to confirm the association between both food intake and allergic rhinitis from a clinical perspective. Our findings suggest that attention should be paid to controlling cheese, cheese, starch, and alcohol intake in patients with allergic diseases in a practical clinical setting. In addition, there is a need to harmonize research methods and define dietary diversity in the future in order to explore potential mechanisms for studying the relationship between food intake and allergy. Our current and previous Mendelian randomization studies of food intake May not have been very precise in classifying food intake, perhaps due to the uploading of classification ranges from high-throughput sequencing results in public databases, and in the future it May be possible to refine and rigorize the results of these studies by using high-throughput sequencing results that are more precisely classified. To fully explore the concept of dietary diversity in the field of immunonutrition, we need to (1) identify gaps in knowledge regarding the impact of nutrition on allergic outcomes, (2) study the overall diet that supports the mechanism, and (3) support education, training, and research in this rapidly growing field.

Our study has certain limitations. Firstly, our research samples are exclusively derived from individuals of European descent and May not be representative of other ethnicities. Secondly, during data collection, we were unable to comprehensively capture all immune cell traits and food intake traits. Our study only analyzed results based on over 900 traits. In addition, our categorization of food intake types was not refined enough to cover certain food intake types.

In summary, our analysis results offer new insights into dietary interventions for patients with allergic rhinitis. We provide methods and prospects for altering dietary compositions to prevent and treat allergic rhinitis patients. Furthermore, we offer a new perspective on immune cell characteristics as potential disease biomarkers for allergic rhinitis patients.

## 5 Conclusion

This study provides a comprehensive assessment of the relationship between food intake and allergic rhinitis and the mediating role played by immune cells in it, using Mendelian randomisation. Firstly, we found causal relationships between food intake and allergic rhinitis and demonstrated that this relationship is not actually mediated by immune cells. Secondly, our findings contribute to new protocols for clinical dietary management of allergic rhinitis patients and provide a reference point for the diagnosis of patients with food allergy-induced allergic rhinitis. Finally, our findings provide new perspectives and ideas for dietary interventions for patients with allergic rhinitis, which May be effective in improving patient prognosis and suggesting new directions for future research.

## Data Availability

All raw data from the study were obtained from the GWAS database (https://www.ebi.ac.uk/gwas/). The numbers corresponding to the original datasets are provided in the manuscript and supplementary documents and are freely available to all readers, please contact the corresponding author for further requests.

## Supplementary material

SUPPLEMENTARY TABLE S1 Food intake GWAS ID.

SUPPLEMENTARY TABLE S2 Horizontal multiple validity test.

SUPPLEMENTARY TABLE S3 Heterogeneity test.

SUPPLEMENTARY TABLE S4 Inverse Mendelian randomization analysis of the causal relationship between food intake and rhinitis.

SUPPLEMENTARY TABLE S5 Mendelian randomization analysis of 30 of 731 immune cell types with causal relationships.

SUPPLEMENTARY TABLE S6 Reverse Mendelian randomization analysis of the causal relationship between 30 immune cell types and rhinitis.

SUPPLEMENTARY TABLE S7 Horizontal pleiotropy test for immune cell type and food intake.

SUPPLEMENTARY TABLE S8 Heterogeneity test for immune cell types and food intake.

SUPPLEMENTARY TABLE S9 Abbreviations.

SUPPLEMENTARY FIGURE S1 Leave-one-out(LOO) analysis of food intake and rhinitis.
