# Supplementary material for: Immunocytes do not mediate food intake and the causal relationship with allergic rhinitis: a comprehensive Mendelian randomization
Source: Front Nutr. 2024 Sep 27;11:1432283. doi: 10.3389/fnut.2024.1432283 (PMC11466801; doi:10.3389/fnut.2024.1432283)
Supplement: Supplementary file 1 [file Data_Sheet_1.zip › STROBE checklist.pdf]

STROBE-MR checklist of recommended items to address in reports of Mendelian randomization studies[1 2]

Item No.	Section	Checklist item	Relevant text from manuscript
1	**TITLE and ABSTRACT**	Indicate Mendelian raabstract if that is a main purpose of the studyndomization (MR) as the study's design in the title and/or the	Immunocytes do not mediate food intake and the causal relationship with allergic rhinitis: A comprehensive Mendelian randomization.
	**INTRODUCTION**		
2	**Background**	Explain the scientific background and rationale for the reported study. What is the exposure? Is a potential causal relationship between exposure and outcome plausible? Justify why MR is a helpful method to address the study question	Observational studies indicate a correlation between food intake and allergic rhinitis. The potential interplay between the immune system and allergic rhinitis might contribute causally to both food intake and allergic rhinitis, providing promising therapeutic avenues. However, elucidating the causal relationship and immune-mediated mechanisms between food intake and allergic rhinitis remains a pending task.
3	**Objectives**	State specific objectives clearly, including pre-specified causal hypotheses (if any). State that MR is a method that, under specific assumptions, intends to estimate causal effects	We utilized a two-sample Mendelian randomization (MR) methodology to explore the causal relationship between food intake and allergic rhinitis. Furthermore, we investigated the potential causal relationship of immune cell signals with allergic rhinitis, as well as the potential causal relationship between food intake and immune cell signals.
	**METHODS**		
4	**Study design and data sources**	Present key elements of the study design early in the article. Consider including a table listing sources of data for all phases of the study. For each data source contributing to the analysis, describe the following:	
		a) Setting: Describe the study design and the underlying population, if possible. Describe the setting, locations, and relevant dates, including periods of recruitment, exposure, follow-up, and data collection, when available.	Immunity-wide GWAS data<br>Summary statistics for each immunophenotype are publicly available in the GWAS Catalog, ranging from accession numbers GCST90001391 to GCST90002121. These statistics cover a total of 731 immunophenotypes, including absolute cell counts (n=118), median fluorescence intensity (MFI) reflecting surface antigen levels (n=389), morphological parameters (MP) (n=32), and relative cell counts (n=192). These features span various developmental stages and cell types of immune cells. The original GWAS for immunophenotypes utilized data from 3,757 European individuals across non-overlapping cohorts.

		The instrumental variable (IV) significance level for each immunophenotype was set at 1 × 10^(-5). We pruned these SNPs using a linkage disequilibrium (LD) r2 threshold of <0.1 within a 500 kb distance.<br>Food intake GWAS data<br>Summary statistics for each food intake phenotype can be accessed publicly in the GWAS Catalog. The significance level for each instrumental variable (IV) was set at 1 × 10^(-5). We pruned these SNPs using a linkage disequilibrium (LD) r2 threshold of <0.001 within a 10,000 kb distance. The GWAS ID corresponding to each of the 171 food intake phenotypes can be found in Table (Table.S1).<br>Allergic Rhinitis GWAS data<br>The Allergic Rhinitis GWAS data were sourced from the public GWAS Catalog, with the GWAS ID being ebi-a-GCST90018792. We pruned these SNPs using a linkage disequilibrium (LD) r2 threshold of <0.001 within a 10,000 kb distance.
	b) Participants: Give the eligibility criteria, and the sources and methods of selection of participants. Report the sample size, and whether any power or sample size calculations were carried out prior to the main analysis	GWAS summary statistics for each immune trait are publicly available from the GWAS Catalog (accession numbers from GCST0001391 to GCST0002121) . A total of 731 immunophenotypes including absolute cell counts (n = 118), median fluorescence intensities reflecting surface antigen levels (n = 389), morphological parameters (n = 32) and relative cell counts (n = 192) were included. Specifically, the MFI, AC and RC features contain B cells, CDCs, mature stages of T cells, monocytes, myeloid cells, TBNK (T cells, B cells, natural killer cells), and Treg panels, while the MP feature contains CDC and TBNK panels. The original GWAS on immune traits was performed using data from 3,757 European individuals and there was no overlapping cohorts. Data for AR were derived from the results of European human genetic sequencing data from the latest release of the GWAS database (ID:ebi-a-GCST90018792) in 2021, containing 24,184,340 SNPs in 4,387 cases and 471,273 controls.Summary statistics for each food intake phenotype can be accessed publicly in the GWAS Catalog. The GWAS ID corresponding to each of the 171 food intake phenotypes can be found in Table (Table.S1 in manuscript).

		c)	Describe measurement, quality control and selection of genetic variants	The instrumental variable (IV) significance level for each immunophenotype was set at 1 × 10^(-5). We pruned these SNPs using a linkage disequilibrium (LD) r2 threshold of <0.1 within a 500 kb distance.
		d)	For each exposure, outcome, and other relevant variables, describe methods of assessment and diagnostic criteria for diseases	The diagnostic criteria for AR are based on ICD-9 and ICD-10 criteria and are derived from the results of European human gene sequencing data from the latest release of the GWAS database (ID:ebi-a-GCST90018792) in the year 2021, details of food intake are detailed in Supplementary Table 1.
		e)	Provide details of ethics committee approval and participant informed consent, if relevant	Only publicly available data were used in this study and details of the source of the data and dataset numbers are provided in the manuscript. The final population source for the data obtained was all European populations, the relevant data were taken from the published GWAS database and informed consent for the study was obtained for the original study, therefore this part of the study did not involve ethics committee approval.
5	**Assumptions**		Explicitly state the three core IV assumptions for the main analysis (relevance, independence and exclusion restriction) as well assumptions for any additional or sensitivity analysis	In the MR analysis, SNPs were considered as IVs. These IVs needed to satisfy three core assumptions: the hypothesis of correlation, the hypothesis of exclusivity, and the assumption of Independence. The first assumption establishes a robust link between SNPs and the variable of exposure. Secondly, the selected SNPs were ensured to have no association with any confounding factors that could influence the relationship between exposure and outcome. Lastly, the SNPs were confirmed to only impact the outcome through exposure factors.
6	**Statistical methods: main analysis**		Describe statistical methods and statistics used	
		a)	Describe how quantitative variables were handled in the analyses (i.e., scale, units, model)	This research does not involve any transformations of quantitative variables.

	b) Describe how genetic variants were handled in the analyses and, if applicable, how their weights were selected	Three analysis methods were employed in this study: Inverse variance weighting (IVW), MR-Egger, and weighted median (WM). The IVW method, considered the primary method for assessing causality, yielded a nominally significantly correlated result when the P value was less than 0.05. To ensure the robustness of the MR results, both MR-Egger and WM methods were employed as complementary approaches. The Cochran's Q test was used to estimate the heterogeneity of SNPs. Additionally, to ensure the reliability of the results, a leave-one-out analysis was carried out. To identify horizontal pleiotropy, the MR-egger intercept was utilized. Causality was evaluated using the odds ratio (OR) and 95% confidence interval (CI).
	c) Describe the MR estimator (e.g. two-stage least squares, Wald ratio) and related statistics. Detail the included covariates and, in case of two-sample MR, whether the same covariate set was used for adjustment in the two samples	Genetic associations with all exposures were taken from a large meta-analysis of GWAS, we obtained SNP-specific Wald estimates and then used inverse variance weighting (IVW) with multiplicative random effects, MR-Egger, and weighted median (WM). The IVW method is a classical method for MR analysis, where the weighted average is calculated by taking the reciprocal of the variance of each IV as the weight, ensuring the effectiveness of all IVs. MR-Egger utilizes a weighted linear regression analysis, providing robust estimates that are independent of the validity of instrumental variables. Nevertheless, it is crucial to acknowledge that these estimates may have lower statistical precision and can be influenced by outlier genetic variation. On the other hand, The problem of estimation accuracy variability is tackled by the WM approach. In a manner reminiscent of the IVW approach, the WM method assigns inverse weights that are contingent upon the variance of individual genetic variants, demonstrating reliability even when causal effects are violated.
	d) Explain how missing data were addressed	In this MR analysis, the issue of missing data was not involved.
	e) If applicable, indicate how multiple testing was addressed	In this MR analysis, multiple exposures or multiple outcomes were not involved, so multiple testing was not performe.

7	**Assessment of assumptions**	Describe any methods or prior knowledge used to assess the assumptions or justify their validity	To assess the risk of weak instrument bias, the selected IVs were assessed for the weak IV bias by calculating the F-statistic.The F-statistic for each SNP was calculated using the formula $F = R^2 (N - K - 1) / [K (1 - R^2)]$. To investigate the degree of bias in the initial causal estimates due to pleiotropic effects, we used some sensitivity analyses, for example: MR-Egger, WM approach. MR-Egger and WM approach were implemented using the R package TwoSampleMR. ”
8	**Sensitivity analyses and additional analyses**	Describe any sensitivity analyses or additional analyses performed (e.g. comparison of effect estimates from different approaches, independent replication, bias analytic techniques, validation of instruments, simulations)	To ensure the robustness of the MR results, both MR-Egger and WM methods were employed as complementary approaches. MR-Egger utilizes a weighted linear regression analysis, providing robust estimates that are independent of the validity of instrumental variables. Nevertheless, it is crucial to acknowledge that these estimates may have lower statistical precision and can be influenced by outlier genetic variation. On the other hand, The problem of estimation accuracy variability is tackled by the WM approach. In a manner reminiscent of the IVW approach, the WM method assigns inverse weights that are contingent upon the variance of individual genetic variants, demonstrating reliability even when causal effects are violated. The Cochran's Q test was used to estimate the heterogeneity of SNPs. Additionally, to ensure the reliability of the results, a leave-one-out analysis was carried out. This analysis aimed to remove SNPs that could have potentially extreme effects. To identify horizontal pleiotropy, the MR-egger intercept was utilized.
9	**Software and pre-registration**		
	a)	Name statistical software and package(s), including version and settings used	All analyses were conducted using R version 4.2.1, with the software packages 'Two-SampleMR' and 'MR-PRESSO'. To visualize the MR analysis, forest plots, scatter plots, and leave-one-out plots were generated using the data analysis function of the Rstudio platform.
	b)	State whether the study protocol and details were pre-registered (as well as when	This study was not pre-registered with the study protocol

RESULTS

10	**Descriptive data**		
		a) Report the numbers of individuals at each stage of included studies and reasons for exclusion. Consider use of a flow diagram	The original GWAS for immunophenotypes utilized data from 3,757 European individuals across non-overlapping cohorts. The instrumental variable (IV) significance level for each immunophenotype was set at 1 × 10^(-5). We pruned these SNPs using a linkage disequilibrium (LD) r2 threshold of <0.1 within a 500 kb distance. Summary statistics for each food intake phenotype can be accessed publicly in the GWAS Catalog. The significance level for each instrumental variable (IV) was set at 1 × 10^(-5). We pruned these SNPs using a linkage disequilibrium (LD) r2 threshold of <0.001 within a 10,000 kb distance. The Allergic Rhinitis GWAS data were sourced from the public GWAS Catalog, with the GWAS ID being ebi-a-GCST90018792. We pruned these SNPs using a linkage disequilibrium (LD) r2 threshold of <0.001 within a 10,000 kb distance.
		b) Report summary statistics for phenotypic exposure(s), outcome(s), and other relevant variables (e.g. means, SDs, proportions)	Summary data on exposures and outcomes are provided in Supplementary Table (1 - 2).
		c) If the data sources include meta-analyses of previous studies, provide the assessments of heterogeneity across these studies	The Cochran's Q test was used to estimate the heterogeneity of SNPs, detailed data are provided in Supplementary Figures.
		d) For two-sample MR:<br>i. Provide justification of the similarity of the genetic variant-exposure associations between the exposure and outcome samples<br>ii. Provide information on the number of individuals who overlap between the exposure and outcome studies	The data presented in this study were derived exclusively from European population samples. These samples were obtained from independent GWAS databases, ensuring minimal overlap and bias.
11	**Main results**		
		a) Report the associations between genetic variant and exposure, and between genetic variant and outcome, preferably on an interpretable scale	The risk of HCC increased with high levels of cathepsin G (IVW: p = 0.029, odds ratio (OR)= 1.369, 95% confidence interval (CI) = 1.033-1.814). Similarly, BTC was associated with elevated cathepsin B levels (IVW: p = 0.025, OR = 1.693, 95% CI = 1.070-2.681). Conversely, a reduction in PCa risk was associated with increased cathepsin H levels (IVW: p = 0.027, OR = 0.896, 95% CI = 0.812-0.988). Lastly, high levels of cathepsin L2 were found to lower the risk of CRC (IVW: p = 0.034, OR = 0.814, 95% CI = 0.674-0.985).
		b) Report MR estimates of the relationship between exposure and outcome, and the measures of uncertainty from the MR analysis, on an interpretable scale, such as	Mengelian randomization estimation reports are detailed in Figures 1-3.

			odds ratio or relative risk per SD difference	
		c)	If relevant, consider translating estimates of relative risk into absolute risk for a meaningful time period	The calculation of absolute risk is detailed in Table 1, Supplementary Tables (4-8).
		d)	Consider plots to visualize results (e.g. forest plot, scatterplot of associations between genetic variants and outcome versus between genetic variants and exposure)	The results are visualized in Figure (1-3).
12	**Assessment of assumptions**			
		a)	Report the assessment of the validity of the assumptions	Firstly, we selected the SNPs of cathepsin as instrumental variables, which have a strong association with digestive system tumors, allowing us to perform Mendelian randomization inferences, and the large F statistics indicate that these analyzes will not be affected by weak instrument bias. Secondly, the selected SNPs were ensured to have no association with any confounding factors that could influence the relationship between exposure and outcome. Lastly, the SNPs were confirmed to only impact the outcome through exposure factors.
		b)	Report any additional statistics (e.g., assessments of heterogeneity across genetic variants, such as I^2, Q statistic or E-value)	The Cochran's Q test did not detect any heterogeneity of the SNPs, These causal relationships did not show any directional pleiotropy according to the MR-Egger intercept test.
13	**Sensitivity analyses and additional analyses**			
		a)	Report any sensitivity analyses to assess the robustness of the main results to violations of the assumptions	Horizontal pleiotropy was assessed using the MR Egger method. The P-values for the MR-Egger regression intercepts were all greater than 0.05, indicating no evidence of horizontal pleiotropy (Table S2).
		b)	Report results from other sensitivity analyses or additional analyses	The LOO analysis revealed a consistent trend for all SNPs included in our analysis, and scatter plots further demonstrated the robustness of our study results (Figure S1).
		c)	Report any assessment of direction of causal relationship (e.g., bidirectional MR)	We primarily utilized the IVW method, and the results indicated causal associations between allergic rhinitis and 7 types of food intake.

				To investigate whether immune cell phenotypes have a causal relationship with allergic rhinitis, we conducted a two-sample Mendelian randomization analysis with 731 immune cell phenotypes as exposures and allergic rhinitis as the outcome. The results revealed that 30 immune cell phenotypes were causally associated with allergic rhinitis (P-value < 0.05) (Table S5).<br>Furthermore, we conducted multivariable Mendelian randomization analysis, which further corroborated that immune cells do not serve as intermediaries in mediating the causal relationship between food intake and allergic rhinitis (Figure 3)
		d)	When relevant, report and compare with estimates from non-MR analyses	This study does not involve non-MR studies.
		e)	Consider additional plots to visualize results (e.g., leave-one-out analyses)	To visualize the MR analysis, forest plots, scatter plots, and leave-one-out plots were generated using the data analysis function of the Rstudio platform, as detailed in FIGURES.
	**DISCUSSION**			
14	**Key results**		Summarize key results with reference to study objectives	To our knowledge, we are the first to explore the causal relationships between immune phenotypes and allergic rhinitis, as well as between food intake and allergic rhinitis. We also investigated the potential mediating effects. For the first time, we incorporated over 900 traits into Mendelian randomization analyses. Furthermore, we identified seven types of food intake and 30 immune cell phenotypes that have causal relationships with allergic rhinitis. However, immune cells do not act as mediators in the causal relationship between food intake and allergic rhinitis.
15	**Limitations**		Discuss limitations of the study, taking into account the validity of the IV assumptions, other sources of potential bias, and imprecision. Discuss both direction and magnitude of any potential bias and any efforts to address them	Our study has certain limitations. Firstly, our research samples are exclusively derived from individuals of European descent and may not be representative of other ethnicities. Secondly, during data collection, we were unable to comprehensively capture all immune cell traits and food intake traits. Our study only analyzed results based on over 900 traits. In addition, our categorization of food intake types was not refined enough to cover certain food intake types.
16	**Interpretation**			
		a)	Meaning: Give a cautious overall interpretation of results in the context of their limitations and in comparison with other studies	The results demonstrate immunocytes do not mediate food intake and the causal relationship with allergic rhinitis.

b) Mechanism: Discuss underlying biological mechanisms that could drive a potential causal relationship between the investigated exposure and the outcome, and whether the gene-environment equivalence assumption is reasonable. Use causal language carefully, clarifying that IV estimates may provide causal effects only under certain assumptions

We also examined the causal relationship between immune cells and allergic rhinitis, revealing that a lower Plasma Blast-Plasma Cell % lymphocyte ratio is associated with a reduced risk of developing allergic rhinitis. Similarly, a lower Absolute Count of CD25+ CD45RA+ CD4 non-regulatory T cells is also associated with a reduced risk. Furthermore, cxcr3 chemokines secreted by T cells play a crucial role in allergic rhinitis , particularly by disrupting the Th1/Th2 balance . Notably, CXCR3 expression in T cells is reduced in allergic rhinitis patients at the onset of the disease. Additionally, Naive CD8+ T cell Absolute Count and Naive CD8+ T cell %CD8+ T cell are protective factors against allergic rhinitis. Previous research has demonstrated that CD8 Tregs can mitigate or suppress the inflammatory response in allergic rhinitis. Furthermore, several T cell subsets and their Absolute Counts, such as Terminally Differentiated CD4-CD8- T cell %T cell, CD28+ CD45RA+ CD8dim T cell %CD8dim T cell, Terminally Differentiated CD4-CD8 - T cell Absolute Count, and CD28+ CD45RA+ CD8+ T cell Absolute Count, are also protective against allergic rhinitis. Our findings also indicate that certain CD molecules expressed on cells, including CD20 on CD20- CD38- B cells, CD27 on CD24+ CD27+ B cells, CD27 on T cells, CD27 on IgD+ CD38- unswitched memory B cells, CD27 on unswitched memory B cells, CD27 on switched memory B cells, CD3 on HLA DR+ CD4+ T cells, CD25 on CD39+ resting CD4 regulatory T cells, CD33 on CD33+ HLA DR+ CD14dim, CD33 on CD33dim HLA DR+ CD11b-, CD33 on Granulocytic Myeloid-Derived Suppressor Cells, CD39 on CD39+ activated CD4 regulatory T cells, CD4 on secreting CD4 regulatory T cells, CD4 on activated & secreting CD4 regulatory T cells, CD45RA on resting CD4 regulatory T cells, and CD45RA on CD39+ resting CD4 regulatory T cells, among others, can delay the onset of allergic rhinitis. For instance, a study conducted by Shiteng Duan in 2019 demonstrated that CD33 recruitment can attenuate IgE-mediated allergic reactions and desensitize mast cells to allergens [107], thereby slowing down the progression of allergic rhinitis. Another study revealed that adhesion facilitates the differentiation of allergic rhinitis CD4IL4 T cells through ICAM1 and E-Selectin [108], leading to the production of the anti-inflammatory factor IL4. Additionally, cell surface protein molecules like HVEM expressed on naive CD8+ T cells also serve as protective factors against allergic rhinitis. A previous investigation showed that the HVEM-NFκB pathway can effectively suppress airway smooth muscle (ASM) proliferation and inflammatory responses by modulating LIGHT (also known as TNFSF14, which mediates signaling that can lead to various inflammatory diseases and airway remodeling)

.However, on the contrary, we have also identified some risk cell factors for allergic rhinitis. For example, Transitional B cell lymphocyte has been associated with an increased risk of allergic rhinitis. The primary role of B cells in allergy is the production of IgE, an antibody isoform that triggers an immediate hypersensitivity reaction via a mediator released by mast cells and basophils . In other words, B lymphocytes can produce allergen-specific IgE antibodies that mediate allergic rhinitis . The results also showed that Granulocyte Absolute Count is one of the risk factors for allergic rhinitis. It has been shown that granulocyte-macrophage colony-stimulating factor (GM-CSF) is a potent pro-inflammatory cytokine, which acts as an eosinophil colony-stimulating factor involved in the onset of allergic rhinitis. In addition, some surface molecules have been validated to promote allergic rhinitis flare-ups, such as CD19 on IgD- CD27- B cell, CD25 on IgD+ CD38- B cell. CD19 is a B-cell specific cell surface molecule belonging to the immunoglobulin superfamily, which is expressed exclusively on B cells. It plays a key role in both B cell activation and autoimmunity . Furthermore, B cells expressing CD19 and CD25 can spontaneously secrete IgA, IgG, and IgM subclasses and exhibit enhanced migratory capabilities. Additionally, these cells secrete elevated levels of pro-inflammatory cytokines, including IL-6 and INF-γ, and are more effective at presenting alloantigens to CD4 T cells . Additionally, CD14 on CD14+ CD16+ monocytes further contributes to the development of allergic rhinitis. Previous research has demonstrated that CD14+ monocytes directly participate in attracting other immune cells to produce pro-inflammatory chemokines and are rapidly recruited to the site of attack.

Our study found that food intake contributes to the development of allergic rhinitis, especially ultra-processed/dairy foods (Figure 1), and this association does not appear to be directly mediated by immune cells as we routinely understand. Excessive intake of ultra-processed/dairy foods increases intestinal permeability, leading to intrinsic mucosal damage and impairment of the epithelial barrier . Studies have shown that the food emulsifier glyceryl monolaurate has been shown to impair intestinal barrier function, leading to dysbiosis of the intestinal flora. Similar findings have been found with emulsifiers such as carboxymethyl cellulose (CMC), which perhaps increases the chance of bacterial invasion and colonisation by damaging the epithelial barrier of the gut. In addition, high levels of ultra-processed food consumption have also been shown to negatively affect the composition and specific functions of the gut microbiome through changes in gut microbial taxa . Dysbiosis of the intestinal

			flora, on the one hand, will cause the bacteria colonising the intestinal tract to stimulate the human immune system with ligands such as lipopolysaccharides, flagellin and fatty acids, which will activate the immune system and lead to the activation of naïve T-cells and the production of Th1, Th2, and Th17 , which will in turn affect the mast cells and other cells, which will produce degranulation changes that will lead to an increase in the production of IgE, thus leading to the onset and progression of allergic rhinitis. On the other hand Watts and Zhu et al by comparing the composition of the gut flora of AR patients with that of the normal population in an analytical study confirmed that the diversity of the gut microbiota was significantly reduced in patients with AR, with an increase in the abundance of pathogenic bacteria such as Anaplasma phylum, and a decrease in the levels of Clostridium and Aspergillus species, and a similar finding was found in the study by liu et al. More importantly, intestinal epithelial cells play a crucial role in intestinal immunity as mediators linking the human immune system and colonising bacteria. Immune cells such as dendritic cells and Tregs in the lamina propria of intestinal epithelial cells react with bacteria colonising the gut. When dysbiosis occurs in the gut causing disruption of the epithelial barrier, bacteria promote the secretion of anti-inflammatory IL-10 by macrophages, decrease mTOR kinase activity and increase the production of antimicrobial peptides. Dendritic cells can ingest invading bacteria and undergo further antigen presentation, recruiting cytokines to maintain the function of Tregs further affecting IL-4 and IFN -γ levels . All of these contribute to the onset and development of upper airway inflammation by regulating the Th1/Th2 balance, and we therefore speculate that perhaps food intake-induced allergic rhinitis may be attributable to dysbiosis of the intestinal flora.We expect that future studies will give more consideration to the mediating role of gut flora in food intake-related allergic rhinitis.
		c) Clinical relevance: Discuss whether the results have clinical or public policy relevance, and to what extent they inform effect sizes of possible interventions	Our analysis results offer new insights into dietary interventions for patients with allergic rhinitis. We provide methods and prospects for altering dietary compositions to prevent and treat allergic rhinitis patients. Furthermore, we offer a new perspective on immune cell characteristics as potential disease biomarkers for allergic rhinitis patients.
17	**Generalizability**	Discuss the generalizability of the study results (a) to other populations, (b) across other exposure periods/timings, and (c) across other levels of exposure	This study comprehensively analyzed the causal relationship between food intake and allergic rhinitis and explored the role of immune cells. Furthermore, the study was limited to a European population, raising questions about its generalizability to other populations.

	**OTHER INFORMATION**		
18	**Funding**	Describe sources of funding and the role offunders in the present study and, if applicable, sources of funding for the databases and original study or studies on which the present study is based	This work was supported by the National Natural Science Foundation of China (No. 82060186).
19	**Data and data sharing**	Provide the data used to perform all analyses or report where and how the data can be accessed, and reference these sources in the article. Provide the statistical code needed to reproduce the results in the article, or report whether the code is publicly accessible and if so, where	Details are provided in the data availability statement and supplementary materials.
20	**Conflicts of Interest**	All authors should declare all potential conflicts of interest	The authors declare that the research was conducted in the absence of any commercial or financial relationships that could be construed as a potential conflict of interest.

This checklist is copyrighted by the Equator Network under the Creative Commons Attribution 3.0 Unported (CC BY 3.0) license.

1. Skrivankova VW, Richmond RC, Woolf BAR, Yarmolinsky J, Davies NM, Swanson SA, et al. Strengthening the Reporting of Observational Studies in Epidemiology using Mendelian Randomization (STROBE-MR) Statement. JAMA. 2021;under review.
2. Skrivankova VW, Richmond RC, Woolf BAR, Davies NM, Swanson SA, VanderWeele TJ, et al. Strengthening the Reporting of Observational Studies in Epidemiology using Mendelian Randomisation (STROBE-MR): Explanation and Elaboration. BMJ. 2021;375:n2233.
