# Supplementary material for: Immunocytes do not mediate food intake and the causal relationship with allergic rhinitis: a comprehensive Mendelian randomization
Source: Front Nutr. 2024 Sep 27;11:1432283. doi: 10.3389/fnut.2024.1432283 (PMC11466801; doi:10.3389/fnut.2024.1432283)
Supplement: Supplementary file 1 [file Data_Sheet_1.zip › Supplementary Table 9.docx]

**Supplementary Table 9: Abbreviations**

| ABBREVIATE | FULL NAME |
| --- | --- |
| MR | Mendelian randomization |
| AR | Allergic rhinitis |
| DCs | Dendritic cells |
| NK cells | Natural killer cells |
| Th 17 | T helper cell 17 |
| TGF-β | Transforming growth factor-β |
| MFI | Median fluorescence intensity |
| MP | Morphological parameters |
| IV | Instrumental variable |
| LD | linkage disequilibrium |
| IVW | Inverse variance-weighted |
| GWAS | [Genome-wide association studies](https://www.nature.com/articles/s43586-021-00056-9) |
| SNP | Single nucleotide polymorphism |
