# Supplementary figures and images for: Immunocytes do not mediate food intake and the causal relationship with allergic rhinitis: a comprehensive Mendelian randomization
Source: Front Nutr. 2024 Sep 27;11:1432283. doi: 10.3389/fnut.2024.1432283 (PMC11466801; doi:10.3389/fnut.2024.1432283)

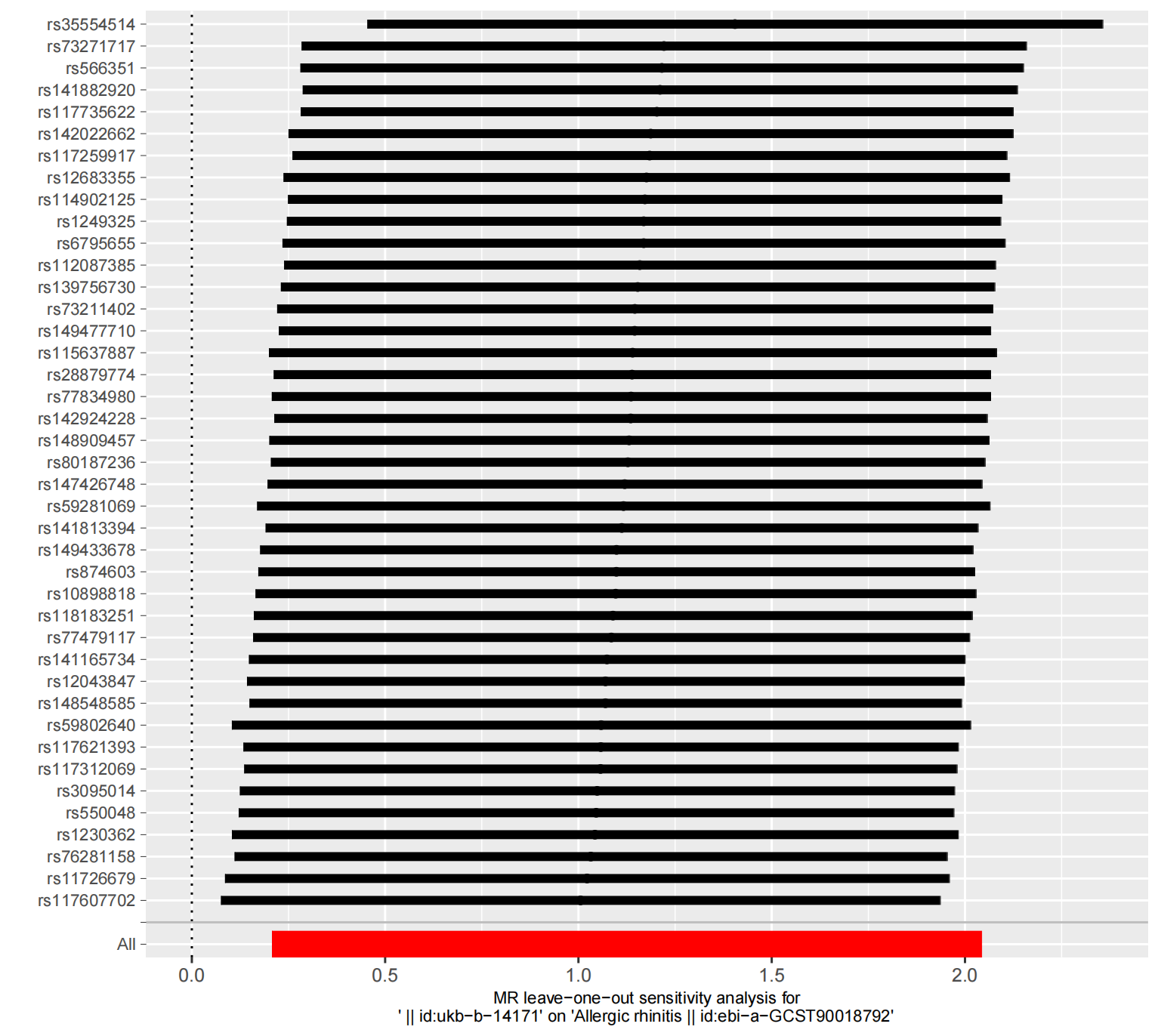

Supplement: Supplementary file 1 [file Data_Sheet_1.zip › Supplementary Image 1.TIF]

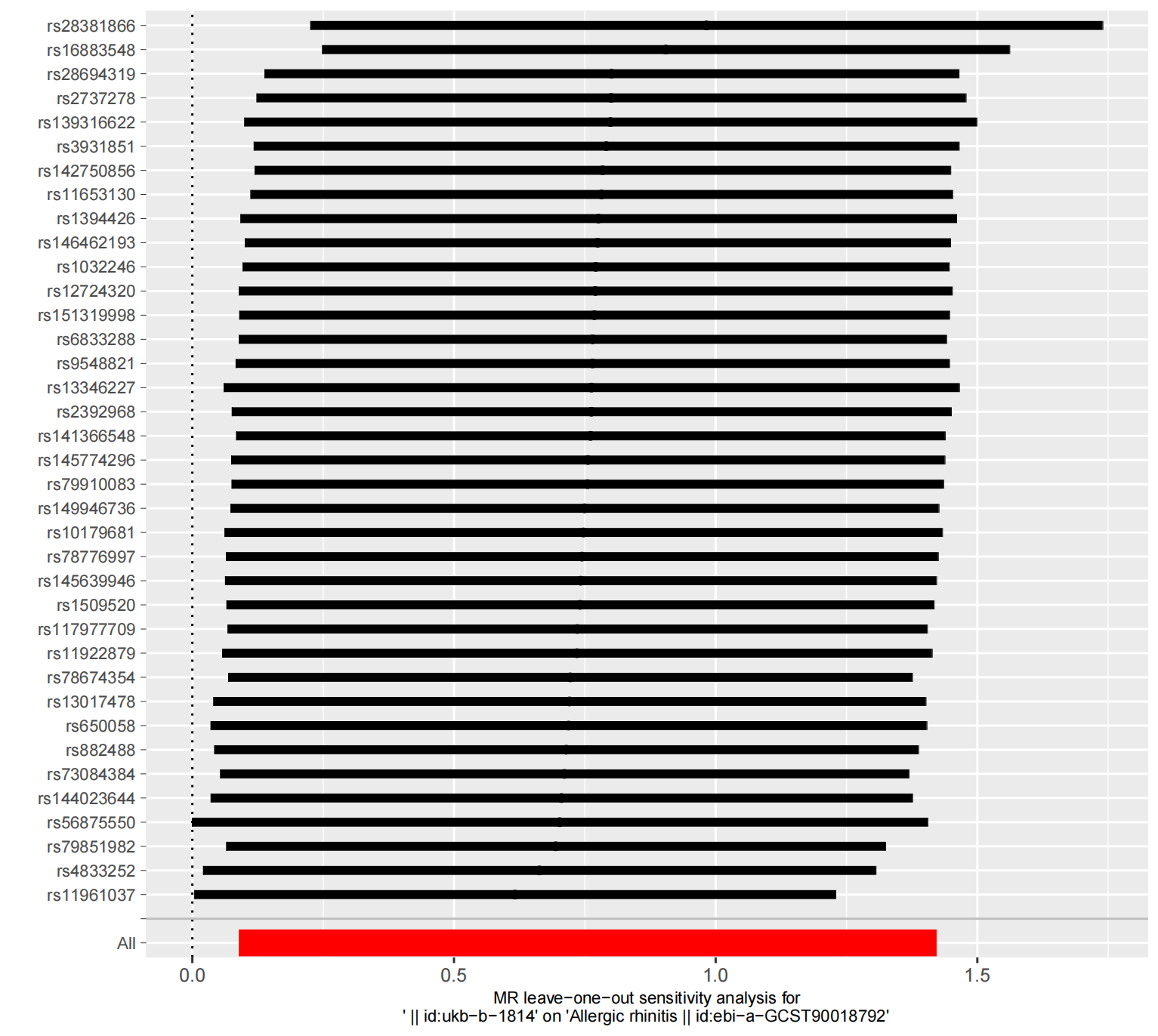

Supplement: Supplementary file 1 [file Data_Sheet_1.zip › Supplementary Image 2.TIF]

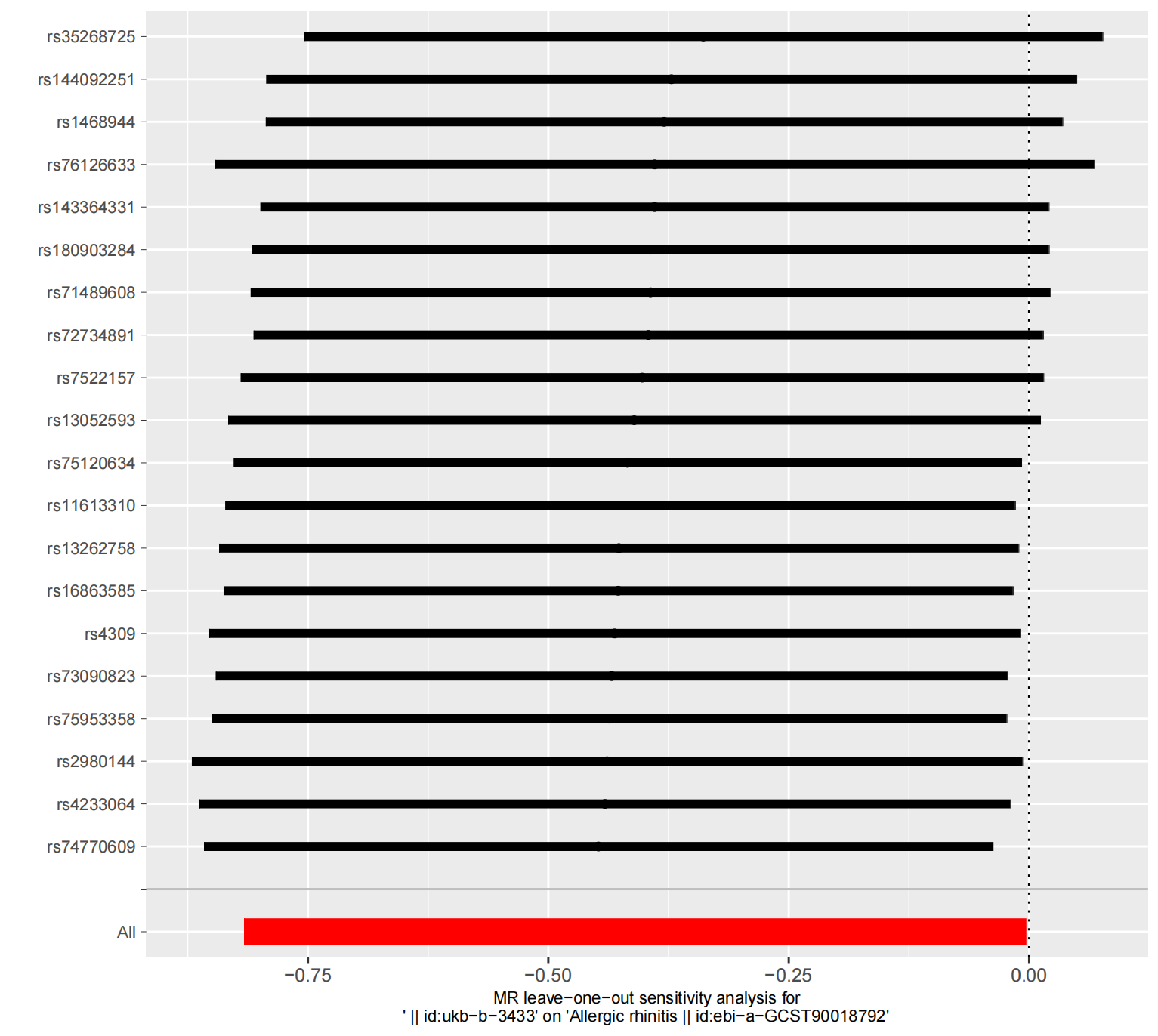

Supplement: Supplementary file 1 [file Data_Sheet_1.zip › Supplementary Image 3.TIF]

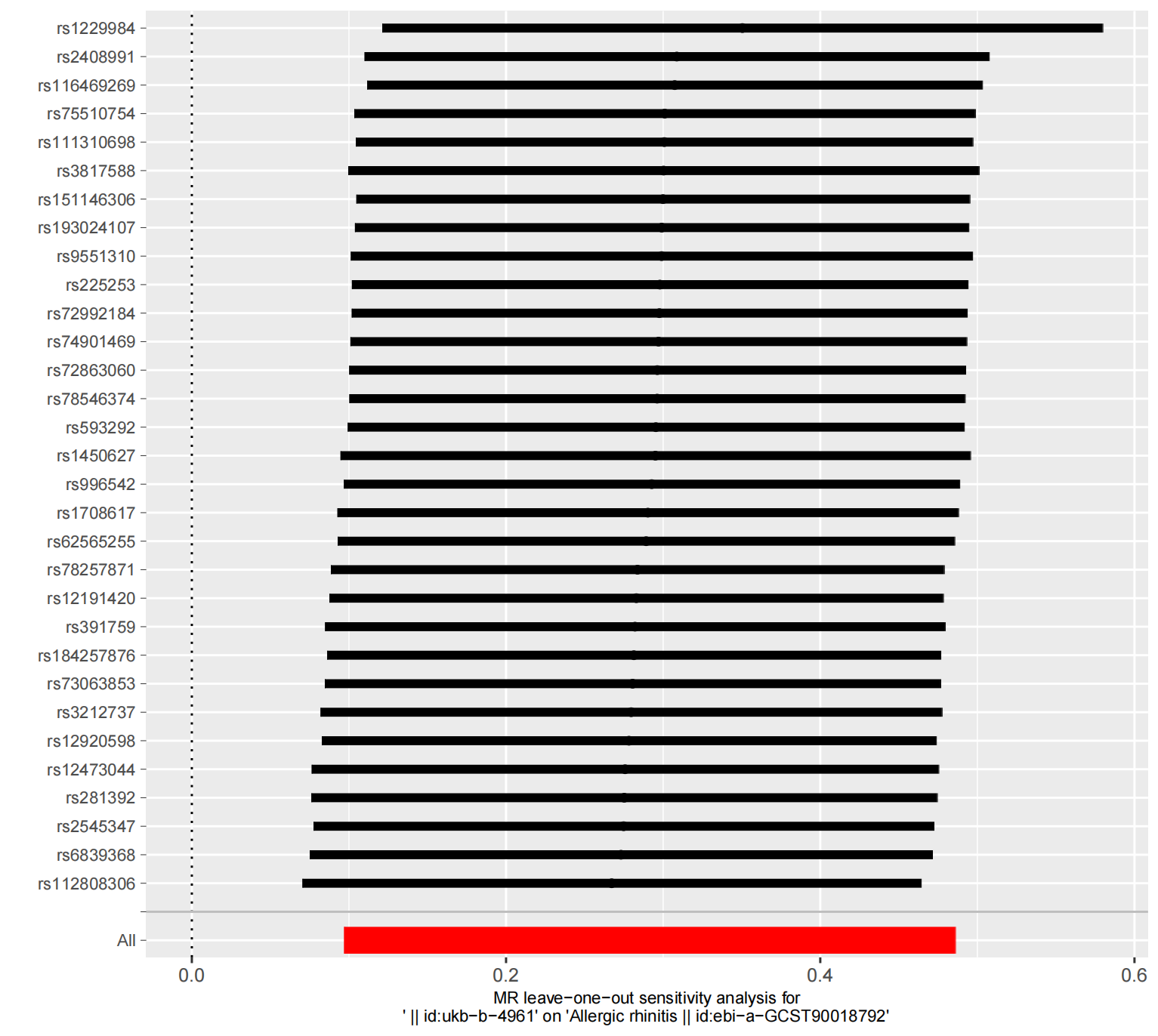

Supplement: Supplementary file 1 [file Data_Sheet_1.zip › Supplementary Image 4.TIF]

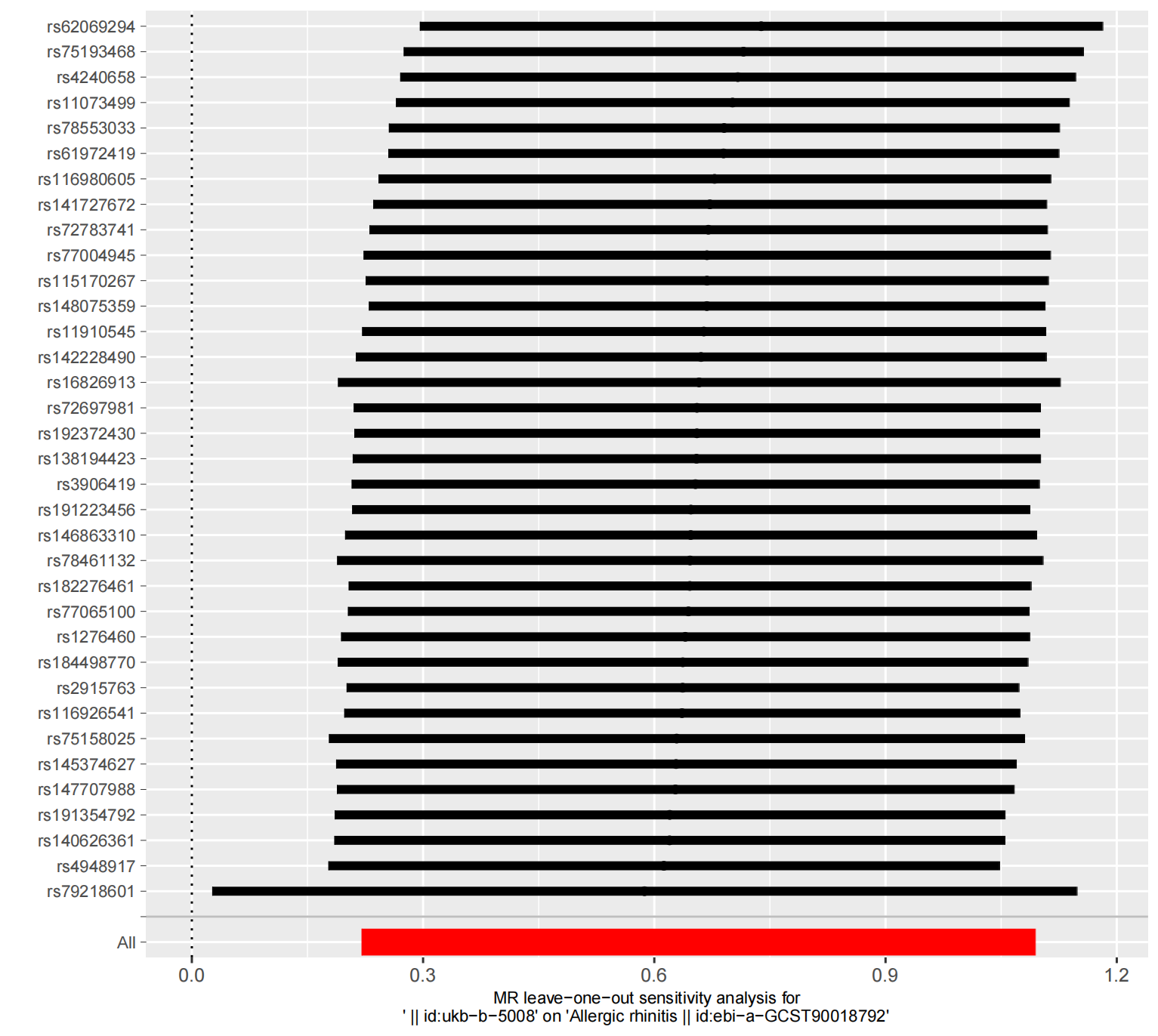

Supplement: Supplementary file 1 [file Data_Sheet_1.zip › Supplementary Image 5.TIF]

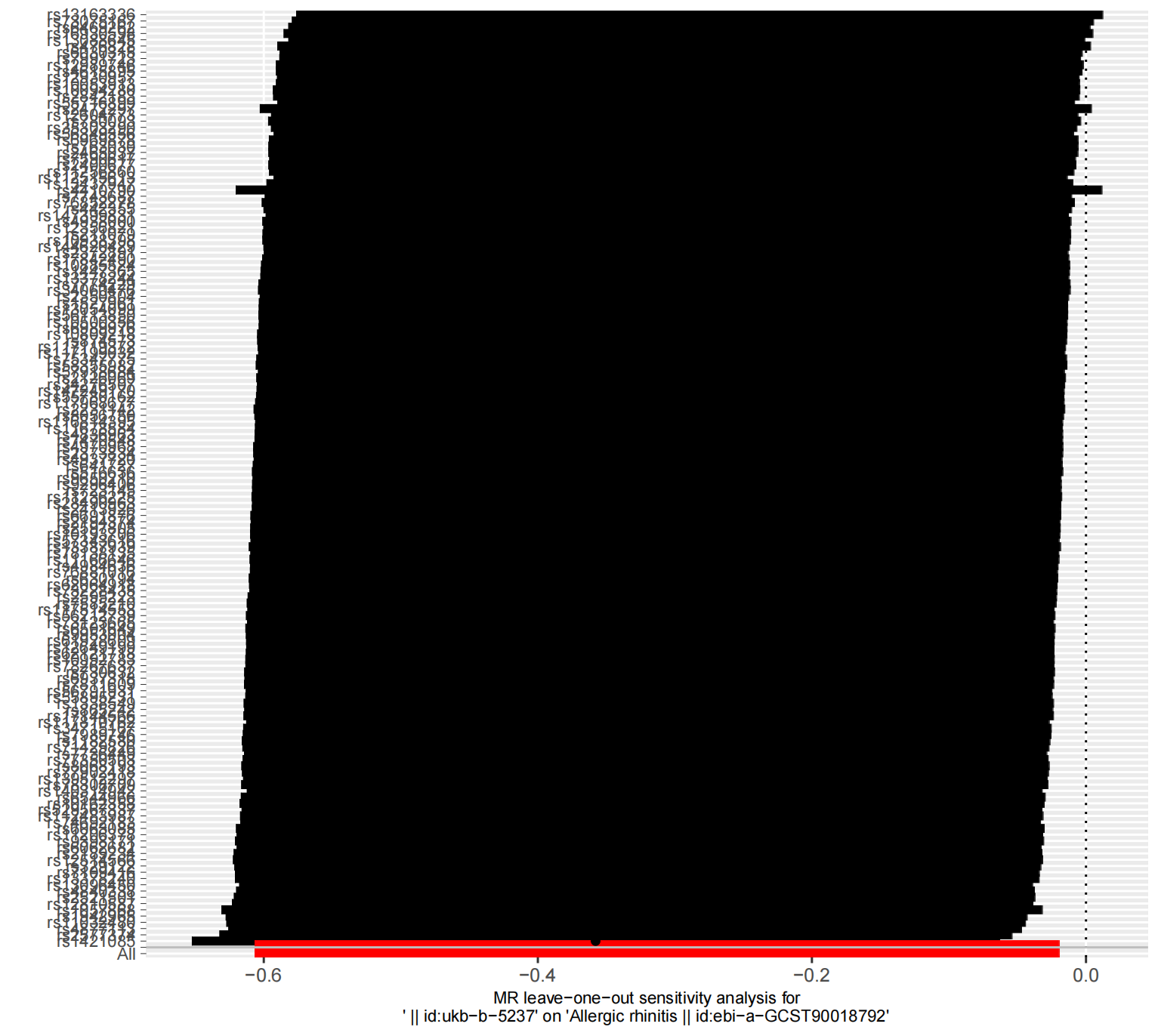

Supplement: Supplementary file 1 [file Data_Sheet_1.zip › Supplementary Image 6.TIF]

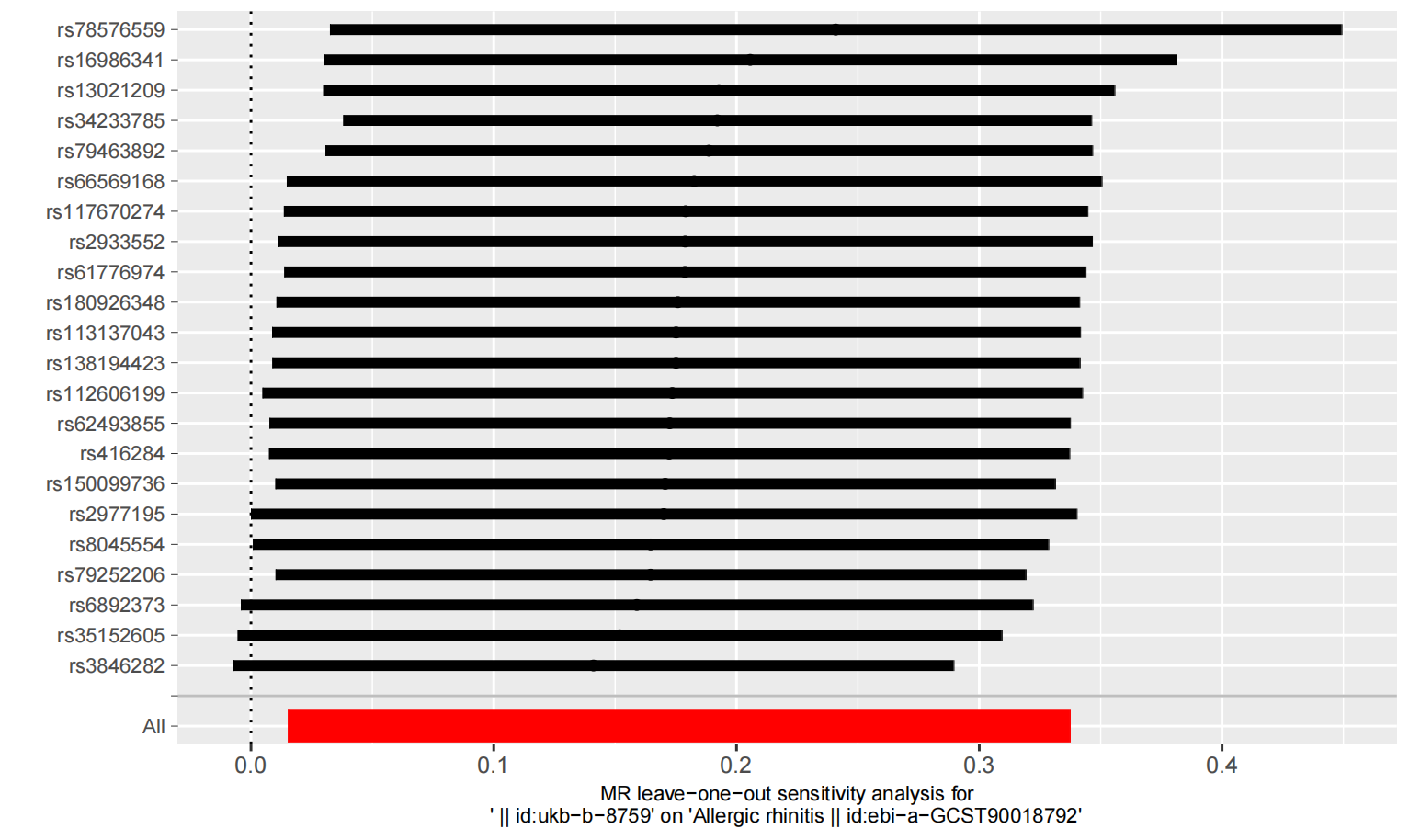

Supplement: Supplementary file 1 [file Data_Sheet_1.zip › Supplementary Image 7.TIF]
